# Transvalvular Precision: Digital Cholangioscopy‐Guided SEMS Deployment for Malignant Ileocecal Obstruction

**DOI:** 10.1111/den.70039

**Published:** 2025-09-18

**Authors:** Shanbin Wu, Yan Zhang, Guoliang Zhao

**Affiliations:** ^1^ Department of Gastroenterology The First Affiliated Hospital of Shandong First Medical University Jinan Shandong China

**Keywords:** cholangioscopy, intestinal obstruction, self‐expandable metal stents

## Abstract

Watch a video of this article.

Self‐expanding metallic stents (SEMS) are a well‐established treatment for acute malignant colonic obstructions, serving as palliative care for unresectable tumors or as a bridge to elective surgery, thereby avoiding emergency enterostomy [1]. While their efficacy in left‐colon malignancies is well documented, their use in proximal colonic applications, particularly for ileocecal stenosis, remains underreported. The technical challenges of placing stents in the ileocecal region arise from anatomical complexities: the greater distance from the anus, the acute angulation at the ileocecal valve (ICV), and tumor‐induced luminal distortion, all contributing to higher single‐attempt failure rates [2, 3]. This report introduces a novel cholangioscopy‐assisted technique that successfully achieved precise stent placement across the ICV in a patient with a malignant ileocecal tumor‐induced intestinal obstruction (Video S1). A 61‐year‐old male presented with acute abdominal pain. CT imaging revealed an ileocecal tumor with suspected ileal intussusception, proximal small bowel dilation (Figure 1), and liver/lung metastases. Following multidisciplinary consultation, palliative SEMS placement was selected. Initial colonoscopy (CF‐HQ290i, Olympus, Japan) demonstrated a circumferential, cauliflower‐like tumor with complete ICV destruction and severe luminal stenosis. Conventional guidewire navigation using a bowie knife failed to traverse the tumor. The subsequent use of an ultra‐slim cholangioscope (VedVision, Vedkang Medical, China; outer diameter 3.1 mm) allowed direct visualization and stricture traversal. After advancing the guidewire through the cholangioscope's working channel, a 10‐cm uncovered SEMS was successfully deployed under dual endoscopic and fluoroscopic guidance. For cases where conventional stent placement is hindered by the ileocecal region's anatomical complexity or severe stenosis, the ultra‐slim cholangioscope reliably traverses strictures under direct visualization. This method provides real‐time intraluminal guidance for precise guidewire advancement and stent deployment, significantly enhancing procedural success rates and avoiding complications associated with blind manipulation. It broadens access to stent‐based interventions, offering a viable bridging strategy for patients unsuitable for immediate or emergency surgery.

**FIGURE 1:**
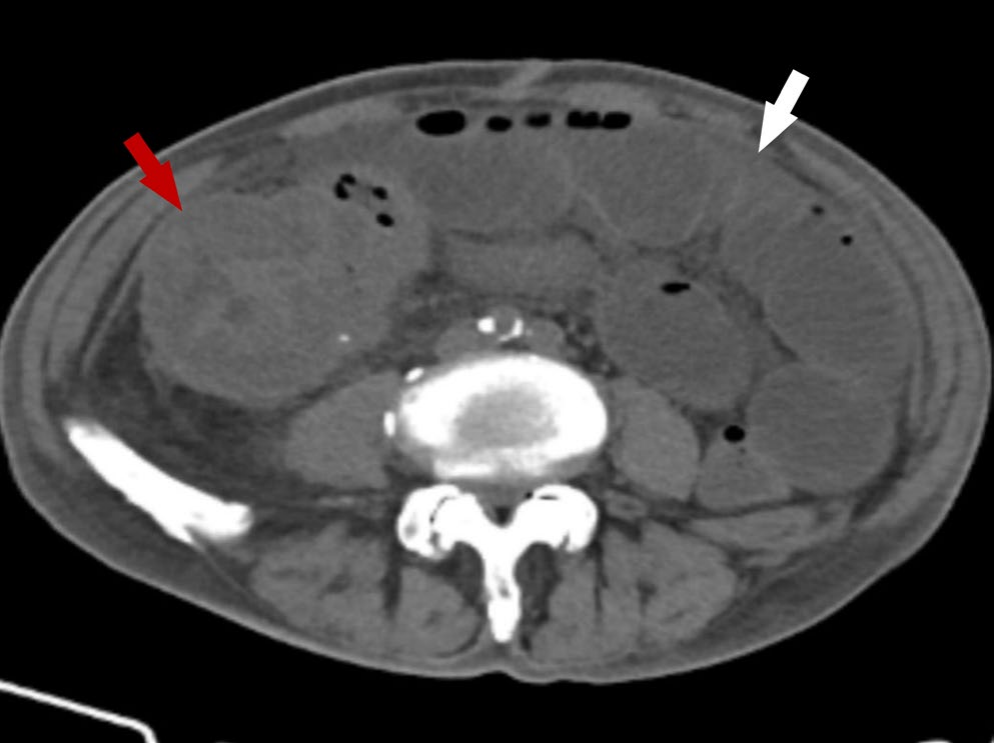
Preoperative computed tomography (CT) shows wall thickening in the ileocecal region and ascending colon (red arrowhead) with luminal narrowing, accompanied by proximal intestinal dilatation (white arrowhead).

## Author Contributions

Shanbin Wu: original draft and video editing. Yan Zhang: review and editing. Guoliang Zhao: surgical operator and supervisory guidance.

## Conflicts of Interest

The authors declare no conflicts of interest.

## Supporting information


**Video S1:** After conventional methods failed, the guidewire successfully traversed the ileocecal valve into the ileum under direct visualization with a cholangioscope, enabling successful metal stent placement.
